# *Clostridioides difficile* Immunity During Pregnancy and Passive Antibody Transfer to Neonates from Cord Blood and Breast Milk

**DOI:** 10.3390/toxins18020111

**Published:** 2026-02-20

**Authors:** Alban Le Monnier, Claire de Curraize, Valérie Seffer, Michel R. Popoff, Pierre Panel, Anne Collignon, Marie-Lise Gougeon

**Affiliations:** 1 Service de Biologie, Unité de Microbiologie, Centre Hospitalier de Versailles, 78150 Le Chesnay-Rocquencourt, France; 2 Service de Microbiologie Clinique, Hôpitaux Saint-Joseph & Marie-Lannelongue, 75014 Paris, France; 3 Institut Micalis UMR 1319, Université Paris-Saclay, INRAE, AgroParisTech, 91400 Orsay, France; 4 Unité Immunité Innée et Virus, Institut Pasteur, 75724 Paris Cedex 15, France; 5 Unité des Toxines Bactériennes, Institut Pasteur, Université Paris Cité, CNRS UMR 2001 INSERM U1306, F-75015 Paris, France; 6 Service de Gynécologie-Obstétrique, Centre Hospitalier de Versailles, 78150 Le Chesnay-Rocquencourt, France; 7 Service de Microbiologie, Hôpital Jean Verdier, 93140 Bondy, France

**Keywords:** *Clostridioides difficile*, pregnancy, newborn, IgM, IgG and IgA antibody responses

## Abstract

Passive transplacental immunity is crucial for neonatal protection from infections. Following *Clostridioides difficile* (*C. difficile*) infection, infants do not develop disease, although *C. difficile* colonization is highly prevalent in infants. This work aimed to characterize humoral immunity specific to *C. difficile* toxins TcdA and TcdB and to surface proteins FliD and Cwp84, well-known colonizing factors, in pregnant women and their neonates. Anti-*C. difficile* antibodies were measured in maternal serum, cord blood, and breast milk from 58 healthy pregnant women and their newborns, enrolled in a prospective study, using a quantitative ELISA. Anti-*C. difficile* antibodies were also measured in pregnant women with *C. difficile* infection (CDI) in a retrospective peripartum case series. We found a high seroprevalence of IgG specific to the four antigens in healthy pregnant women, regardless of colonization by *C. difficile*. However, pregnant women exhibited lower concentrations of TcdA-specific IgG antibodies compared to age-matched non-pregnant women. A strong positive correlation between maternal and cord blood IgG specific to TcdA, TcdB, FliD, and Cwp84 was observed, suggesting a transplacental transfer of *C. difficile*-specific IgG antibodies to neonates. In breast milk, a high seroprevalence of IgA specific to the two toxins was detected, and positive correlations between maternal serum and breast milk antibody levels highlight a preferential transfer of TcdB-specific IgG and Cwp84-specific IgG to breast milk, providing the infant with a protective barrier against *C. difficile*. Lastly, since pregnant women are at increased risk for *C. difficile* infection (CDI), we characterized the specific antibody response in a retrospective peripartum case series. Sera from peripartum women with CDI exhibited similar median concentrations of TcdA, TcdB, FliD, and Cwp84 IgM and IgG to those of healthy pregnant women. Moreover, except for one case, antibody concentrations remained stable during the longitudinal evolution of *C. difficile* response before and after diagnosis of CDI, without any booster effect. Altogether, these data are consistent with antibody-mediated maternal protection of neonates from *C. difficile*-associated disease. Larger studies exploring immune factors involved in protection from *C. difficile*-associated disease during pregnancy are needed.

## 1. Introduction

*Clostridioides difficile* (*C. difficile*) is an anaerobic Gram-positive spore-forming bacterium, now recognized as a major cause of community-associated and healthcare-associated gastrointestinal infections, particularly in adults and elderly patients [1,2]. Symptoms of *C. difficile* infection (CDI) range from diarrhea, fever, nausea, and abdominal pain. Complications may include pseudomembranous colitis, toxic megacolon, and sepsis. CDI represents a major public health burden due to its morbidity, mortality, and economic impact [3,4,5,6]. Although CDI is well described, some individuals may be colonized without developing symptoms [7,8].

Contamination by *C. difficile* occurs with ingestion of spores. *C. difficile* spores germinate in vegetative cells, colonize the gut, multiply, and release toxins, leading to intestinal symptoms [9]. Antibiotic-induced gut dysbiosis is critical for the digestive colonization process, removing protective commensal bacteria. In addition, several surface components are involved in the colonization step, and among them are the protease Cwp84 and the cap protein FliD [10,11].

Pregnancy, as well as the antepartum and postpartum periods, puts women at an increased risk of developing CDI, largely due to major risk factors such as prior antibiotic exposure and hospitalization [12,13]. A three-fold increase in the incidence of peripartum CDI from 1997 to 2017 was reported in the US [14]. In a retrospective study performed on cases of peripartum CDI in six French hospital centres between 2008 and 2013, most of the cases had been exposed to at least one key risk factor of CDI: antibiotic use and/or hospitalization. The postpartum cases often had a cesarean section. Complications, such as pseudomembranous colitis, toxic megacolon, or death, were reported [15]. Another study also reported that CDI was associated with significant maternal morbidity (sepsis, paralytic ileus, venous thromboembolism) and maternal death [16]. Moreover, women with peripartum CDI are generally more likely to undergo cesarean delivery [12,14,15]. Two additional factors contribute to an increased risk of peripartum CDI. The first one is the unavoidable exposure of these women to toxigenic *C. difficile* strains of asymptomatic infant carriers [17]. Indeed, Rousseau *et al*. reported that pathogenic *C. difficile* strains circulate in asymptomatic infants from the community, who represent a potential reservoir of pathogenic strains [17]. The second one is a state of temporary immune suppression normally observed during a healthy pregnancy [17,18]. Indeed, pregnant women are considered immunocompromised hosts by virtue of immune adaptation to their allogeneic fetus. A Th1-dominant microenvironment becomes biassed towards the Th2 phenotype to enable immunological tolerance that is necessary for pregnancy to continue. This Th1/Th2 shift reduces the production of antibodies against *C. difficile* TcdA and TcdB, which may influence CDI risk [19].

Both innate and adaptive immune responses contribute to protection against CDI. The adaptive response against *C. difficile* is characterized by antibodies against TcdA and TcdB toxins and also against non-toxin antigens. Regarding antitoxin antibodies, seroprevalence (i.e., serum IgG) against TcdA and TcdB is relatively widespread in the healthy population. This occurs in the absence of active infection or even colonization, suggesting a persistent long-life exposure to *C. difficile* [20,21,22]. Protection against initial and recurrent *C. difficile* infection requires an effective IgG immune response against *C. difficile* toxins [23,24,25]. While these antibodies do not protect from colonization, they influence disease susceptibility. Kyne et al. reported a greater increase in IgG specific to TcdA in patients who remained asymptomatically colonized compared to patients who progressed to CDI [23]. Regarding a possible protective role of antibodies targeting colonization factors, immunization studies in hamsters with Cwp84 and the flagellar protein FliC administered by the mucosal route were shown to result in a significant decrease in intestinal *C. difficile* colonization [26,27]. Patients with CDI were shown to develop antibodies against FliC, FliD, and Cwp84 during the course of the disease [28,29], but to date, no studies have demonstrated their protective role.

During childhood, asymptomatic intestinal colonization by *C. difficile* is frequent, as reported in a prospective screening of *C. difficile* in the stools of infants aged 0 to 2 years. Molecular typing of the isolates revealed that infants are widely colonized by non-toxigenic strains [30]. Colonization is an early (neonatal) process, and it lasts for several months [17]. Toxigenic isolates can be carried by some infants, thus representing a potential reservoir of pathogenic strains [17,30]. However, infants are protected from disease, even in the presence of toxin-producing strains. A lack of receptors for clostridial toxins and a relative immaturity of the neonatal intestine with a less diverse intestinal microbiota than that of adults may help to protect infants from the deleterious effects of *C. difficile* toxins [31,32,33,34].

Antibodies against *C. difficile* toxins are detected in some infants [20], and a recent study reported that toxigenic *C. difficile* colonization in infants is associated with IgG and IgA antibodies specific to TcdA and TcdB, with an in vitro neutralizing activity of TcdB antibodies [35]. Neonates are protected from most infections through the transfer of maternal antibodies, particularly IgG, across the placenta and, after birth, through breastfeeding [36]. The majority of transplacental transfer of IgG occurs in the last trimester of pregnancy, and the efficiency of this transfer can vary from one antigen-specificity to another [36]. With regard to *C. difficile* infection, the protective effects of maternal immunity in neonates have been demonstrated in animal models, but not in women. Therefore, we assumed that protection of infants from CDI disease results from maternal antibodies passively acquired transplacentally or in breast milk. The characterization of maternal-specific antibody response to *C. difficile* and passive maternal immunity to the neonate remains limited. This study consists of three parts: (1) a prospective observational study of mother–neonate dyads that explored *C. difficile* carriage as well as toxin- and non-toxin-specific antibody responses in pregnant women and their neonates, and also the question of maternal protection from disease through cord blood and breast milk *C. difficile* specific antibodies; (2) a comparison of *C. difficile* humoral immunity between healthy pregnant women and matched non-pregnant women; and (3) a retrospective study for the characterization of *C. difficile*-specific antibody response in pregnant women with CDI.

## 2. Results

### 2.1. Study Population and C. difficile Carriage in the ICD-MATER Cohort

A total of fifty-eight pregnant women have been included in the study. Age ranged from 20 to 41 years (median: 31 years). Forty-three (74.1%) breastfed their child, and only 18 (41%) agreed to give their breast milk. Among the 58 newborns included, 25 were girls, and 33 were boys.

**Figure 1:**
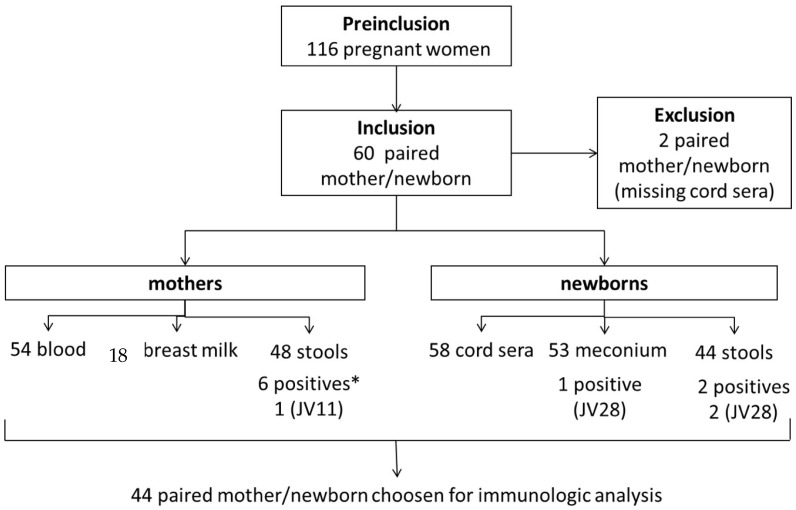
Flowchart of pregnant women and neonates included in the ICD-MATER study. * *C. difficile* carriage in stools and meconium; JV11 and JV28: PCR ribotypes characterized at Jean Verdier Hospital.

Asymptomatic *C. difficile* carriage was explored in meconium from 53 out of 58 newborns, and only one (2%) was positive. *C. difficile* carriage was also explored in the stools, and among the 44 newborns’ stools collected, two (5%) were positive. One of the two newborns was positive for both meconium and stool (Figure 1). None of the isolated strains were toxigenic.

Regarding *C. difficile* carriage by the mothers, six out of the 48 (13%) tested stools were positive. It should be noted that none of the mothers of the two newborn carriers were carriers themselves. Moreover, none of the isolated strains were toxigenic.

Antibiotic treatment was administered during the *peripartum* period for 15 (26%) mother/newborn pairs. Of these, one mother and one newborn were *C. difficile* carriers. Three (5%) newborns received antibiotic treatment at birth, but none of these children were *C. difficile* carriers.

### 2.2. TcdA- and TcdB-Specific Antibodies in Pregnant and Non-Pregnant Women from ICD-MATER Cohort

In order to characterize the seroprevalence of *C. difficile* in pregnant women and age-matched non-pregnant women, we developed a quantitative ELISA assay to assess serum concentrations and isotypes of antibodies specific to *C. difficile* toxin and non-toxin antigens. This immunological study has been achieved on samples from 44 mother/newborn pairs, chosen according to the availability, for all pairs, of cord blood, maternal serum, and stools. We first measured the levels of antibodies against toxins A and B.

**Figure 2:**
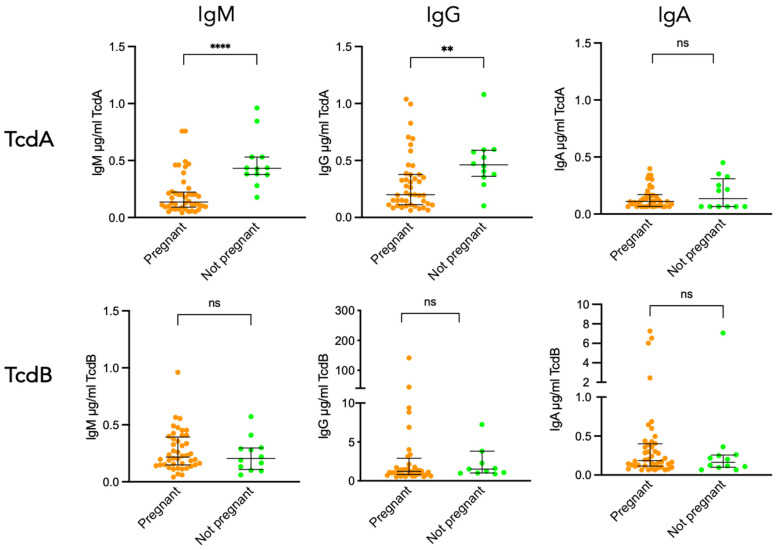
TcdA and TcdB seropositivity in pregnant and non-pregnant women. Comparison of TcdA- and TcdB-specific IgM, IgG, and IgA antibody responses in pregnant women and matched non-pregnant women (*n* = 12). Statistical significance of differences between the two groups was determined using the Mann–Whitney U test. ** *p* < 0.01; **** *p* < 0.0001; ns, not significant.

Figure 2 shows that all sera from pregnant women, and from matched controls as well, exhibited levels of TcdA- and TcdB-specific IgM and IgG antibodies above the detection threshold. In contrast, TcdA-specific IgA was detected in only 25% of sera from pregnant women and 50% from non-pregnant women, while TcdB-specific IgA was detected at a higher frequency, i.e., 91% in pregnant women and 83% in non-pregnant women. Comparison of antibody levels between the two groups revealed significantly lower concentrations of TcdA-specific antibodies in pregnant women. This was observed both for IgM and IgG, but not for IgA. Indeed, the median concentration of IgM against TcdA was significantly lower in pregnant women compared to non-pregnant women (*p* < 0.0001), and the same applied for the median concentration of IgG against TcdA for pregnant women vs. non-pregnant (*p* = 0.005). In contrast, TcdB-specific IgM, IgG, and IgA levels were comparable in pregnant vs. non-pregnant women (Figure 2).

### 2.3. FliD- and Cwp84-Specific Antibodies in Pregnant and Non-Pregnant Women from ICD-MATER Cohort

**Figure 3:**
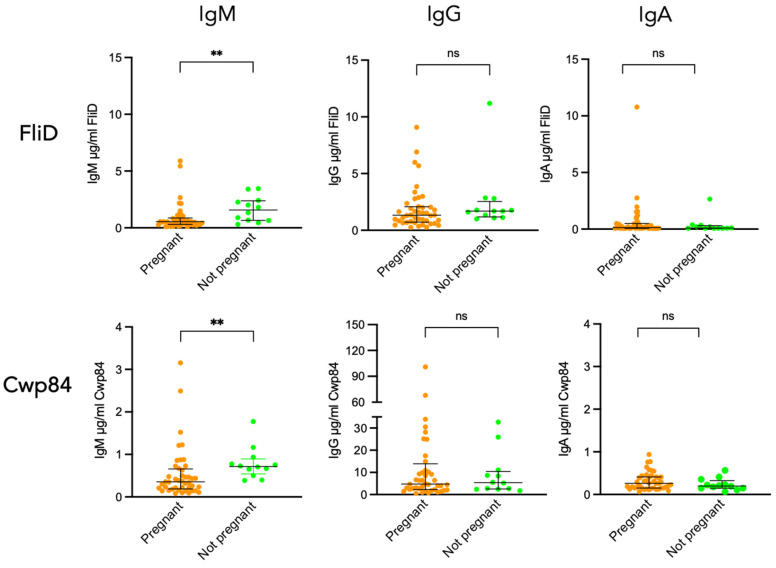
FliD and Cwp84 seropositivity in pregnant and non-pregnant women. Comparison of FliD- and Cwp84-specific IgM, IgG, and IgA antibody responses in pregnant women and matched non-pregnant women. ** *p* < 0.01; ns, not significant.

While *C. difficile* pathogenesis is mainly due to TcdA and TcdB, surface proteins such as the flagellar protein FliD and the protease Cwp84 are involved in the colonization process, the initial step of pathogenesis. Since protection from CDI involves antibodies against these colonization factors [11], the measurement of their concentration is of interest. Figure 3 shows that, as observed for TcdA and TcdB, 100% of sera from pregnant women, and from non-pregnant women as well, exhibited FliD and Cwp84 IgM and IgG antibodies above the detection threshold. Regarding IgA, 100% of sera from pregnant and non-pregnant women had antibodies against Cwp84, while antibodies against FliD were detected in only 66% of sera from both groups.

Comparison of antibody levels between pregnant and non-pregnant women revealed a significantly lower level of IgM against FliD and Cwp84 in pregnant compared to non-pregnant women (*p* = 0.0046 for FliD and *p* = 0.0028 for Cwp84). In contrast, the levels of IgG and IgA specific for these two antigens were similar in sera from pregnant vs. non-pregnant women (Figure 3).

Six out of the 48 pregnant women included in this study were asymptomatic carriers of non-toxigenic strains of *C. difficile.* Therefore, we compared toxin A/B and FliD/Cwp84 IgM, IgG, and IgA responses between non-colonized vs. colonized women. No significant difference was found for all tested antigens and regardless of the isotype.

### 2.4. C. difficile Toxin-Specific Antibodies in Cord Blood, Breast Milk, and Serum in Mother–Newborn Pairs (ICD-MATER)

Despite frequent *C. difficile* colonization in the first months of life, most infants remain asymptomatic [37]. Among several factors that may contribute to protection from disease, maternal antibodies may play a role. To explore this hypothesis further, we investigated cord blood, mothers’ sera, and breast milk for their content in *C. difficile*-specific antibodies in 44 complete mother–newborn pairs. Breast milk was obtained from 18 mothers.

**Figure 4:**
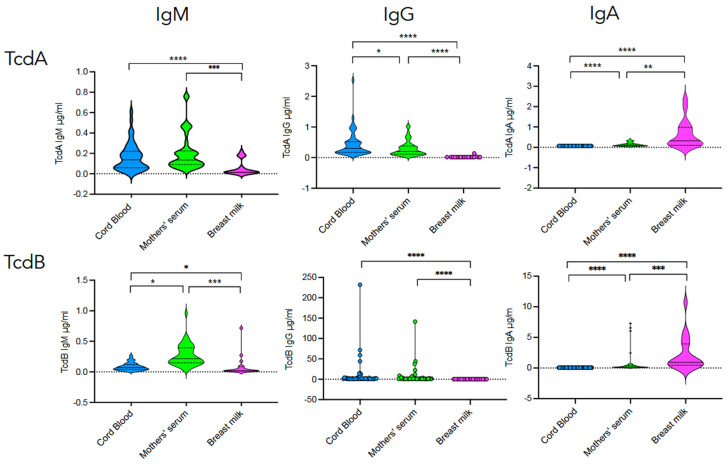
TcdA and TcdB seropositivity in cord blood, mother’s serum, and breast milk. Comparison of TcdA- and TcdB-specific IgM, IgG, and IgA antibody responses in cord blood (*n* = 44), mother’s serum (*n* = 44), and breast milk (*n* = 18). Statistical significance of differences between the groups was determined using the Wilcoxon signed-rank test. * *p* < 0.05; ** *p* < 0.01; *** *p* < 0.001; **** *p* < 0.0001; ns, not significant.

Figure 4 shows that cord blood contains high concentrations of IgG antibodies specific to TcdA and TcdB, while no IgA could be detected. All cord blood samples exhibited detectable levels of TcdA- and TcdB-specific IgG. Very low concentrations of IgM were detected. Comparison of antibody levels between cord blood and mothers’ sera revealed small differences, although significant for toxin-specific IgG.

In breast milk, the vast majority of antibodies detected were IgA. TcdA- and TcdB-specific IgA were detected in 89% and 100% of breast milk samples, respectively. In contrast, toxin-specific IgG was only partially detected in breast milk samples, i.e., 11% (2/18) for TcdA and 33% (6/18) for TcdB. Comparison of toxin-specific antibody levels between breast milk and mothers’ sera highlights an enrichment of breast milk with TcdB-specific IgA (Figure 4).

### 2.5. FliD- and Cwp84-Specific Antibodies in Cord Blood, Breast Milk, and Serum in Mother–Newborn Pairs (ICD-MATER Cohort)

**Figure 5:**
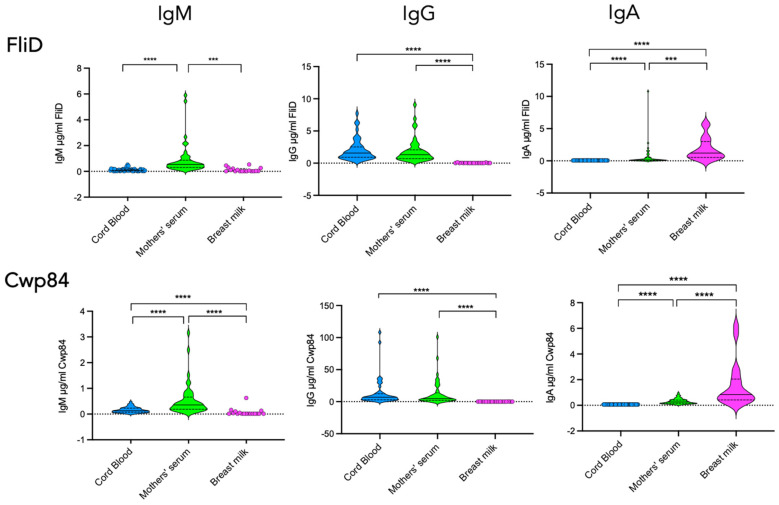
FliD and Cwp84 seropositivity in cord blood, mother’s serum, and breast milk. Comparison of FliD- and Cwp84-specific IgM, IgG, and IgA antibody responses in cord blood (*n* = 44), mother’s serum (*n* = 44), and breast milk (*n* = 18). Statistical significance of differences between the groups was determined using the Wilcoxon signed-rank test. *** *p* < 0.001; **** *p* < 0.0001; ns, not significant.

Figure 5 shows the pattern of antibodies specific to FliD and Cwp84. As expected, cord blood contains mostly IgG antibodies specific to these two antigens, while, as observed for TcdA and TcdB, no IgA could be detected. Comparison of antibody levels between cord blood and mothers’ sera showed comparable levels both for FliD and Cwp84. Regarding IgM responses, their levels against both factors were barely detectable and significantly lower in cord blood compared to mothers’ sera.

In breast milk, as observed for the antibody response against TcdA and TcdB, the vast majority of antibodies were IgA. FliD- and Cwp84-specific IgA were detected in all breast milk samples. In contrast, IgG was only partially detected in these samples, i.e., 28% (5/18) for FliD and 50% (9/18) for Cwp84. No IgM against FliD and Cwp84 was detected in breast milk samples. Comparison of antibody levels between breast milk and mothers’ sera shows an enrichment of breast milk with FliD-specific IgA and Cwp84-specific IgA (Figure 5).

### 2.6. Correlations Between Cord Blood, Maternal Serum, and Breast Milk C. difficile Antibody Levels (ICD-MATER Cohort)

**Figure 6:**
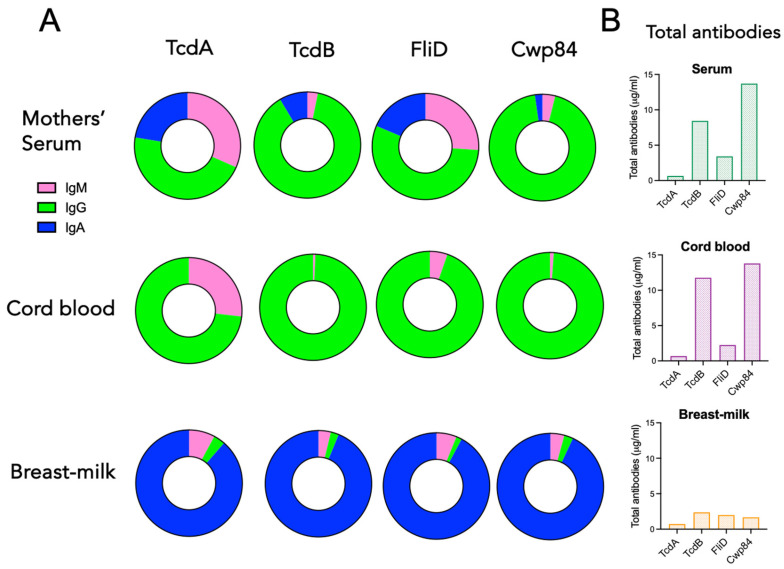
Isotypic composition of *C. difficile* antibody response in cord blood, mothers’ serum, and breast milk. (**A**) IgM, IgG, and IgA isotypic composition of TcdA, TcdB, FliD, and Cwp84 antibodies in cord blood (*n* = 44), mothers’ serum (*n* = 44), and breast milk (*n* = 18). (**B**) Composition of total antibodies from cord blood, mothers’ serum, and breast milk relative to indicated antigenic specificities.

Figure 6A shows the isotypic composition of antibodies against TcdA, TcdB, FliD, and Cwp84 in mothers’ serum, cord blood, and breast milk. Maternal serum and cord blood showed a dominant IgG response against each of the four antigens tested, while breast milk was almost entirely composed of IgA. In breast milk, the total amount of antibodies, irrespective of their isotypes, was very low, with comparable levels against the four antigens (0.5 to 2 µg/mL) (Figure 6B). On the other hand, maternal serum and cord blood showed a substantial level of total antibodies, with a dominant response against TcdB and Cwp84 (8 to 14 µg/mL in maternal serum and 12 to 14 µg/mL in cord blood, respectively), compared to TcdA and FliD (0.5 to 3 µg/mL in maternal serum and 0.5 to 2 µg/mL in cord blood, respectively).

**Figure 7:**
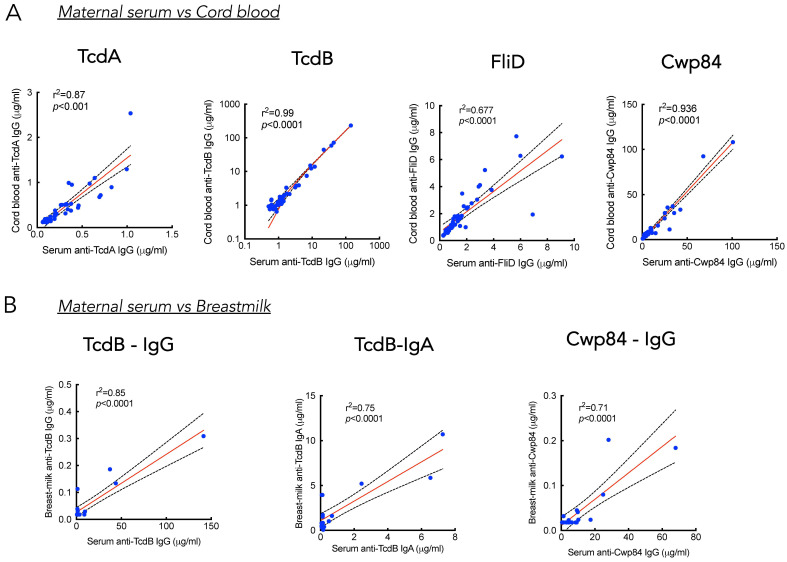
Correlations between maternal serum, cord blood, and breast milk seropositivity. (**A**) Linear regression of correlation between maternal serum and cord blood IgG antibodies specific to TcdA, TcdB, FliD, and Cwp84 (*n* = 44). (**B**) Linear regression of correlation between maternal serum and breast milk IgG specific to TcdB and Cwp84, and IgA specific to TcdB (*n* = 18).

A strong correlation was detected between the levels of maternal IgG and cord blood IgG for each of the four antigens (r^2^ = 0.87 for TcdA, r^2^ = 0.99 for TcdB, r^2^ = 0.68 for FliD, and r^2^ = 0.94 for Cwp84) (Figure 7A), suggesting a maternal transplacental transfer of *C. difficile*-specific IgG antibodies to neonates.

Regarding the IgA response, the question is irrelevant since cord blood does not contain *C. difficile*-specific IgA (Figure 4, Figure 5 and Figure 6). Transmission into breast milk of maternal antibodies against *C. difficile* is suggested by the positive correlation of antibody levels between maternal serum and breast milk for TcdB-specific IgG (r^2^ = 0.85), TcdB-specific IgA (r^2^ = 0.75), and Cwp84-specific IgG (r^2^ = 0.71) (Figure 7B). No correlation between breast milk and serum antibody levels was found for TcdA and FliD antigens. Of note, only two out of 18 milk samples tested contained IgG against TcdA, and six out of 18 contained IgG against FliD.

### 2.7. Antepartum C. difficile Infection Cases

Regarding the fourteen *peripartum* cases we previously described [15], we had access to pre- and post-infection sera for five of them. Clinical and biological characteristics are described in Table 1. The median age was 32 years (range 24 to 36). All women developed symptomatic CDI in the *antepartum* period. All but one infected woman (P5) had antibiotic therapy before CDI onset, all had been hospitalized before the CDI episode, including three for pregnancy with complications (P3, P4, and P7), and one for severe asthma (P1). P5 developed diarrhea before her hospital admission, and CDI was diagnosed only five days later. Two had fever associated with diarrhea, and four developed an inflammatory response (hyperleukocytosis and elevated CRP). Four had obstetric complications such as premature rupture of membranes and gestational diabetes. One infected woman (P3) had an episode of recurrence.

### 2.8. C. difficile-Specific Antibody Response in Pregnant Women with CDI

**Figure 8:**
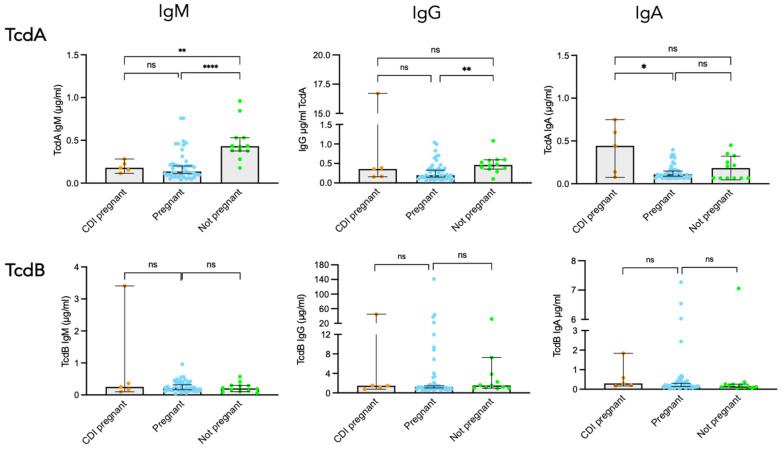
Toxin-specific antibody responses in healthy or CDI pregnant women and healthy non-pregnant women. Comparison of TcdA and TcdB IgM, IgG, and IgA antibody responses in CDI pregnant women, healthy pregnant women, and non-pregnant women. Statistical significance of differences between the three groups was determined using the Mann–Whitney U test. * *p* < 0.05; ** *p* < 0.01; **** *p* < 0.0001; ns, not significant.

In order to characterize the antibody response in pregnant women with CDI, we compared concentration levels of IgM, IgG, and IgA against toxins and colonizing factors between three groups of women: CDI pregnant (*n* = 5), healthy pregnant (ICD-MATER cohort) (*n* = 44), and healthy non-pregnant women (matched controls) (*n* = 12). For pregnant women with CDI, the time-point chosen was the closest after CDI onset, which varied from three to 18 days. Comparative antibody responses to TcdA and TcdB are shown in Figure 8. All sera from pregnant women with CDI exhibited detectable levels of TcdA-specific antibodies. However, surprisingly, the levels of TcdA-specific IgM and IgG were comparable between infected and non-infected pregnant women, with IgM median concentration of 0.18 µg/mL (IQR: 0.13–0.25) vs. 0.13 µg/mL (IQR: 0.09–022), respectively, and IgG median concentration of 0.36 µg/mL (IQR: 0.16–8.5) vs. 0.2 µg/mL (IQR: 0.22–0.37), respectively. Moreover, as already shown above, sera from pregnant women exhibited significantly lower concentrations of IgM and IgG against TcdA compared to non-pregnant women.

Nevertheless, sera from pregnant women after CDI onset exhibited higher TcdA-specific IgA compared to uninfected pregnant women [0.44 µg/mL vs. 0.11 µg/mL, respectively]. Regarding TcdB-specific antibody responses, all sera from pregnant women with CDI exhibited detectable levels of IgM, IgG, and IgA antibodies. However, serum concentrations of all three isotypes were comparable in infected vs. non-infected pregnant women (Figure 8).

**Figure 9:**
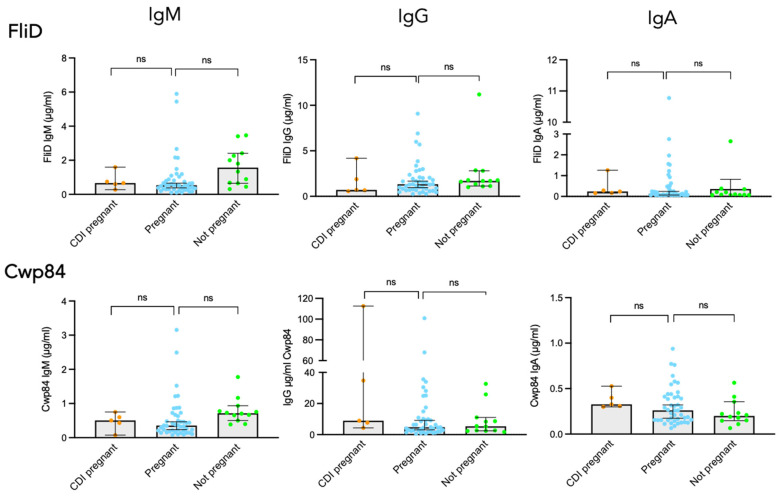
FliD- and Cwp84-specific antibody responses in healthy or CDI pregnant women and healthy non-pregnant women. Comparison of FliD and Cwp84 IgM, IgG, and IgA antibody responses in CDI pregnant women (*n* = 5), healthy pregnant women (*n* = 44), and non-pregnant women (*n* = 12). ns, not significant.

The same comparative analysis was performed for the response to the two colonization factors. Sera from pregnant women with CDI exhibited comparable levels of antibodies against FliD and Cwp84, regardless of the isotype, compared to uninfected pregnant women (Figure 9).

**Figure 10:**
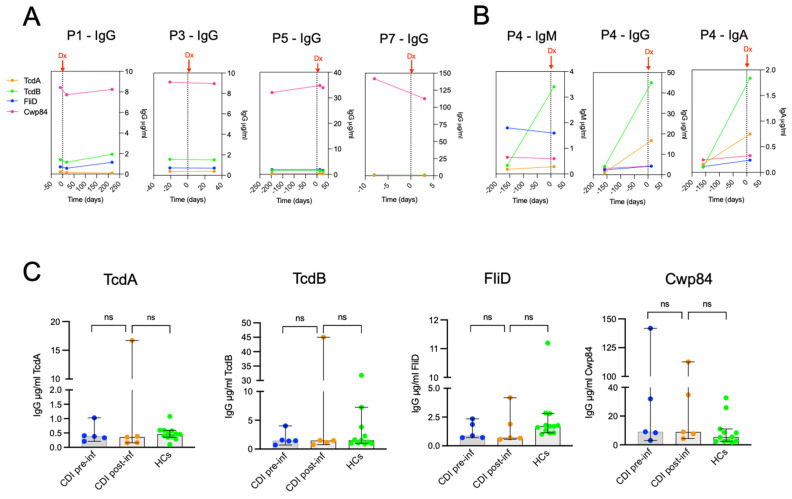
*C. difficile* IgG antibody response pre- and post-infection in pregnant women. (**A**) Evolution of IgG response specific to TcdA, TcdB, FliD, and Cwp84 in available samples before CDI diagnosis (dated from 10 days to 197 days) and post-infection (dated from 3 to 217 days after CDI diagnosis). (**B**) *C. difficile* pre- and post-infection antibody responses in P4. (**C**) Comparison of *C. difficile* antibody responses in pregnant women (*n* = 5) before infection, post-infection, and healthy pregnant women (*n* = 12). Data are shown in a box plot (median with 95% CI). HCs: healthy controls; ns, not significant.

Longitudinal evolution of *C. difficile* IgG antibody response specific to TcdA, TcdB, FliD, and Cwp84 in patients P1, P3, P5, and P7 is shown in Figure 10A. Available pre-infection samples dated from 10 days to 197 days before CDI diagnosis, while available post-infection samples dated from three to 217 days after CDI diagnosis. Strikingly, no increase in IgG specific to TcdA, TcdB, FliD, and Cwp84 was detected following *C. difficile* infection, except for P4, who showed a different pattern (Figure 10B). An induction of TcdA and TcdB-specific IgG and IgA responses was detected, while FliD and Cwp84 antibody responses remained the same.

As shown in Table 1, no obvious clinico-biological feature of P4 could be related to a strong response to TcdB. Comparison of antibody IgG levels between CDI pre-infection vs. CDI post-infection vs. matched non-infected pregnant controls showed no statistical difference for each of the tested antigens (Figure 10C). Same observations were made for IgM and IgA.

## 3. Discussion

Antibody-mediated immunity is crucial for the prevention and treatment of various toxin-related diseases, including CDI. However, knowledge about natural immunity in CDI is still limited [20,38,39], especially in pregnant women [40]. During pregnancy, the maternal immune system evolves to protect developing offspring against pathogens while tolerating a foreign fetus. Immunological changes occurring during pregnancy include a state of immune suppression due to a shift towards Th2-based immunity, a decrease in cellular immunity required to control infections, and a reduction in B cells and IgG levels, especially in the second and third trimesters [41,42,43,44].

We conducted a prospective observational study in 58 mother–neonate dyads (ICD- MATER cohort) with the aim of characterizing humoral immunity against toxins and two surface proteins well-known as colonizing factors for *C. difficile* in pregnant women and their neonates. In addition, maternal serum, cord blood, and breast milk samples were analyzed to investigate maternal transfer of antibodies.

All sera from pregnant women exhibited a complete isotypic pattern of antibodies specific to the toxins TcdA and TcdB, and the two colonizing factors FliD and Cwp84. This high seroprevalence in healthy pregnant women is in agreement with prior studies showing that the majority of healthy adults have detectable antibodies to *C. difficile* in their sera, even in the absence of *C. difficile* colonization or active infection. This natural response is thought to arise from environmental exposure to *C. difficile* in early infancy [20] and perpetuated through adult life colonization [7,20,22,30,45,46]. In the present study, we also analyzed *C. difficile* carriage and found that colonized pregnant women showed similar antibody levels compared to the non-carriers for all the tested antigens and for all three isotypes. In previous studies, asymptomatic carriers of *C. difficile* were reported to exhibit higher serum IgG antibody levels to TcdA compared with non-colonized patients who later developed diarrhea, suggesting a protective role of these antibodies against symptoms [23].

To evaluate the impact of pregnancy on serum concentrations of *C. difficile*-specific antibodies, we also analyzed samples from age-matched non-pregnant women. Strikingly, the only difference detected in antibody responses between these two groups was the lower concentration of TcdA-specific IgM and IgG antibodies in pregnant women. The impact of a reduced natural immunity to TcdA on susceptibility to CDI during pregnancy is difficult to assess, given the scarcity of studies in this field. The question of the potential protective role of toxin-specific antibodies against the occurrence of CDI has been addressed by a number of studies. For example, the rise in IgG antibodies specific to TcdA and TcdB occurring in patients with CDI during the course of illness was not found to be associated with protection against recurrence [39]. In contrast, a correlation between high anti-TcdB titers and protection from recurrence was reported [28]. In addition, naturally occurring anti-TcdB, but not anti-TcdA antibodies, within placebo recipients in phase III studies of an antitoxin human monoclonal antibody [47], were found to be protective against recurring CDI [48]. These findings are consistent with the fact that TcdB is more strongly associated with illness than TcdA in CDI [49,50].

Infants are protected against symptomatic *C. difficile* infection despite a high prevalence of *C. difficile* colonization [30,37]. The reason for this is unknown, but transplacental transfer of maternal IgG antibodies to infants may be involved. The characterization of maternal–neonatal transfer of *C. difficile*-specific antibodies remains limited. Our study shows the prevalence, in cord blood, of an IgG response against the four tested antigens, with a dominant response against TcdB and Cwp84. Moreover, the strong positive correlations we found between maternal and cord blood IgG specific to TcdA and TcdB and the two colonizing factors suggest a transplacental transfer of *C. difficile*-specific IgG antibodies to neonates and imply that passive antibody transfer relies on maternal antibody concentration.

Evolutionarily, in addition to the transfer of antibodies via the placenta, infants receive passive immunity through mucosal antibodies via breast milk [51,52]. Breast milk antibodies confer immunity against mucosal pathogens until the infant is able to mount an immune response [53]. Breastfeeding during the first six months of life is strongly protective against infectious diseases, as documented by Victora et al., reducing mortality among breastfed infants by at least 88% compared to non-breastfed infants [54]. The beneficial effect of breastfeeding was also shown in a study comparing the microbiome of more than 1500 infants from the child cohort according to the breastfeeding status of the infant [55]. Distinct gut microbiomes were observed depending on the breastfeeding status, and *C. difficile* colonization rate was lowest for babies exclusively breastfed compared to partially breastfed and exclusively formula-fed infants [55]. Early studies showed that human milk contains mostly IgA, and colostrum samples collected postpartum were shown to exhibit a neutralizing activity to *C. difficile* TcdA and TcdB, and to protect adult hamsters against a lethal challenge with TcdA [56,57]. These findings suggest that *C. difficile* toxin-specific antibodies are transferred via breast milk to infants, but the precise quality and levels of the antibodies have not yet been explored. In the present study, we report that the vast majority of *C. difficile* antibodies in breast milk were IgA, with a seroprevalence for TcdA and TcdB close to 100%, while toxin-specific IgG was detected in less than 30% of samples. Comparison of toxin-specific antibody levels between breast milk and mothers’ sera highlighted an enrichment of breast milk with TcdA-, TcdB-, FliD-, and Cwp84-specific IgA. Moreover, our data highlight a preferential transfer of TcdB-specific IgG and IgA, and Cwp84-specific IgG to breast milk, as suggested by positive correlations between maternal serum and breast milk antibody levels. These maternal antibodies provide the infant with a protective barrier against *C. difficile*.

Pregnant women are at increased risk for CDI, particularly during the peripartum period [40]. We previously described in detail a retrospective peripartum case series of CDI in France [15]. We took advantage of the availability of serum samples collected during the follow-up in the antepartum CDI cases to perform a comprehensive study of *C. difficile*-specific antibody responses in these women. We report that the only response to be increased in CDI pregnant women was IgA specific to TcdB. These findings are compatible with a lack of increase in IgG against TcdA, TcdB, FliD, and Cwp84 that we observed during the longitudinal evolution of *C. difficile* response before and after diagnosis of CDI. Despite years of study, there remains an incomplete understanding of the antibody response to *C. difficile* pathogenic toxins and other antigens in CDI [21]. Comparison of humoral immunity in initial CDI cases and recurring infections has shown that patients with a single episode of CDI exhibited a stronger antitoxin response than patients with recurrent disease [23,58,59]. The magnitude of anti-TcdA response correlated with resistance to symptomatic infection and protection against recurrence [23,38]. However, other studies reported that protection from recurrence correlated with higher levels of anti-TcdB antibodies [24,60,61]. It has also been suggested that *C. difficile*-associated disease was due to an inability to mount an appropriate immune response [62], as reported in a recent study of acute *C. difficile* infection, where antibody titers specific to TcdA and TcdB remained stable during the first week of CDI diagnosis without any booster effect [63], confirming previous studies [39,61]. This is consistent with our observation on the CDI cases of peripartum women in the present study. These data raise the question of the mechanisms behind this lack of booster effect, and they challenge the hypothesis of paradoxical killing of primed B, central to the development of a protective secondary antibody response by antibody-dependent uptake of toxins, suggested by Eichel-Streiber et al. [61].

Several limitations must be acknowledged. First, the drawbacks associated with mixed study design, i.e., prospective dyads, non-pregnant controls, and a retrospective peripartum CDI subgroup. Second, the small sample size of women with peripartum CDI requires confirmation on a greater number of subjects. Third, the study design for the dyads did not plan a longitudinal follow-up of the infants in order to study neonatal clinical endpoints, such as the occurrence of symptomatic CDI or colonization dynamics.

In conclusion, this study reports a high seroprevalence of *C. difficile* in healthy pregnant women, with variable concentrations of antibodies specific to TcdA and TcdB and to surface proteins FliD and Cwp84, and with comparable levels to those detected in sera from non-pregnant women, except for TcdA-specific IgM and IgA. Transplacental antibody transfer through cord blood of *C. difficile*-specific IgG antibodies to neonates was detected for the four antigens, and a preferential transfer of TcdB-specific IgG and IgA, and Cwp84-specific IgG to breast milk was reported as well. Lastly, sera from peripartum women with CDI exhibited similar concentrations of TcdA, TcdB, FliD, and Cwp84 IgM and IgG to those of healthy pregnant women, in contrast to TcdB-specific IgA, which was increased. Altogether, these data are consistent with maternal transfer to neonates of antibodies specific to *C. difficile*. Larger studies exploring immune factors involved in protection from *C. difficile*-associated disease during pregnancy are needed.

## 4. Methods

### 4.1. Study Design and Populations

#### 4.1.1. ICD-MATER Cohort

Mother–neonate pairs were recruited in the Centre Hospitaller de Versailles (Le Chesnay, France). Between 31 August and 3 November 2011, all pregnant women were informed about the study and pre-included during the last month of pregnancy. Definitive inclusion occurred at the time of delivery.

Pregnant women were eligible for inclusion in the study if they were 18 years of age or older and able to provide informed consent. Exclusion criteria included a positive human immunodeficiency virus test, blood transfusion in the month prior to delivery, and pre-included women who no longer wished to take part in the study on the day of delivery. A secondary exclusion criterion included the absence of cord blood. Standardized questionnaires were used to collect demographic information and medical history during the mother’s hospital admission for childbirth. Matched healthy non-pregnant women (*n* = 12) were recruited in the control group. The study was approved by the Institutional Review Board (*Comité de Protection des Personnes Ile de France XI, n°IRB 11033*).

Serum from age-matched (from 20 to 38 years old) non-pregnant women (*n* = 12) was provided by the Institut Pasteur’s Biological Resources Centre (CRBIP).

#### 4.1.2. *Clostridioides difficile* Infection (CDI) During Pregnancy

The clinical and biological features of these cases were previously reported in detail [15]. *C. difficile* strains that were isolated in culture and frozen were characterized using PCR ribotyping as described by Bidet et al. [30,64]. In addition, PCR with Xpert^®^ *C. difficile* (Cepheid, Maurens-Scopont, France) was used to presumptively identify BI/NAP1/027 strains by specific detection of *tcdC* deletion at nucleotide 117 and/or *cdtA* gene encoding the binary toxin.

#### 4.1.3. ICD-MATER Clinical Samples

Samples were collected from delivery and prior to the mother–neonate discharge home. Cord blood samples were collected at the time of delivery into sodium–heparin via venipuncture of the umbilical cord vessels. Maternal blood samples were collected within two days after delivery during postpartum biological testing performed as part of standard clinical care. Breast milk was collected during the onset of lactation from day 2 to discharge from maternity (day 3 to day 6). Maternal stools were collected in the delivery room or prior to discharge from the maternity ward. Neonate meconium was collected upon issue, and neonate stool samples were collected from diapers just before discharge from the maternity ward. All samples were stored at −80 °C before analysis.

### 4.2. Microbiological Analysis

Stool samples were cultured on multiple selective media. A Columbia agar supplemented with cefoxitin (8 mg/L), cycloserine (250 mg/L), 5% horse blood, and 0.1% sodium taurocholate (CCTa) was freshly prepared in the laboratory, whereas CLO^®^ agar and ChromID™ *Clostridioides difficile* agar were obtained commercially (bioMérieux). Suspected colonies of *C. difficile* were confirmed using MALDI-TOF coupled to mass spectrometry (Bruker Daltonics, Billerica, MA, USA). *C. difficile* strains that have been isolated in culture and frozen were characterized using the *C. diff* Quik Chek Complete^®^ immunochromatographic assay (Alere, Jouy-en-Josas, France) and PCR ribotyping as described by Bidet et al. [30,64].

### 4.3. C. difficile Toxins and Colonization Factors

TcdA and TcdB were produced and purified from *Clostridioides difficile* VPI10463. Toxins were produced by the dialyzing cultivation method with BHI broth (Oxoid, Servilab, Le Mans, France) as outer medium and 10% NaCl as inner medium [65]. Cultures were performed at 37 °C for 4 days. TcdA and TcdB were purified as previously described [62] using ammonium sulphate precipitation, ion exchange chromatography (DEAE-Sephacel, GE Healthcare Life Sciences, Little Chalfont, Buckinghamshire, UK), and gel filtration (Superdex G200, GE Healthcare Life Sciences, Velizy Villacoublay, France).

Recombinant surface protein FliD and Cwp84 involved in the colonization process were produced and purified as previously described by Pechine et al. [28].

### 4.4. Quantitative ELISA for C. difficile-Specific Antibody Response

Maternal sera, cord blood, and breast milk were tested by ELISA for their concentration of IgM, IgG, and IgA specific for *C. difficile* toxins and surface antigens, using a quantitative assay, as we previously described for Shiga toxin stxB [66]. Briefly, 96-well plates (MaxiSorp, Nunc, Dominique Dutscher, Bernolsheim, France) were coated overnight at 37 °C with 100 μL of 0.1 µg/mL TcdA, 0.2 µg/mL TcdB, 0.5 µg/mL FliD, and 0.9 µg/mL Cwp84. After washing, unsaturated sites were blocked with a saturating buffer. The serum to be tested was diluted, added to each well, and incubated for 1 h at 37 °C. After washing, goat anti-human IgG, IgA, or IgM alkaline phosphatase-conjugated antibodies were added, and antigen-specific antibodies were detected with para-nitrophenyl phosphate (pNPP) substrate. The reaction was stopped by 100 μL of sodium hydroxide, and the optical density was read at 405 nm. The calibration curves were set up using purified polyclonal human IgG, IgA, and IgM (Sigma, Chambery, France). The concentrations of serum antibodies were calculated according to the calibration curves. Serum Ig concentrations are expressed in μg/mL. The detection thresholds for IgM, IgG, and IgA concentrations were 0.018 µg/mL, 0.014 µg/mL, and 0.066 µg/mL, respectively.

### 4.5. Statistical Analysis

Statistical analyses were performed using GraphPad Prism (version 10.4.2). Quantitative variables were expressed as median and interquartile range. Paired *t*-tests were used to compare antibody titers in serum, breast milk, and cord blood. Non-parametric Mann–Whitney U tests were used to compare antibody levels in pregnant women, non-pregnant women, women with CDI, and matched controls. Linear regression and Spearman rank correlation were used to analyze correlations between maternal serum seropositivity, cord blood, and breast milk seropositivity. Thresholds for seropositivity for each of the three isotypes are indicated in the description of the quantitative ELISA assay. Corrections for multiple comparisons were not done because the study was focused on planned comparisons. Values of *p* < 0.05 were considered statistically significant.

## Figures and Tables

**Table 1:** Clinical and biological characteristics of antepartum *C. difficile* infection cases.

ID	Age	Indication for Prior Hospitalization	Previous Antibiotic Therapy (Indication)	Cortico-Steroid Therapy	Obstetric or Delivery Complication	Diarrhea with Fever	Inflammatory Syndrome	Treatment	Recurrence	Samples Pre- and Post-CDI	PCR Ribotype
P1	32	Asthma	Tazocillin, ciprofloxacin, cefepime (Pneumonia)	Yes	No	Yes	Yes	Vancomycin	No	Day −10/+18	078
P3	24	Pathologic pregnancy	Amoxicillin (PRM ^a^)	Yes	PRM^a^	Yes	Yes	Metronidazole	Recurrence	Day −21/+31	Not BI/NAP1/0 27
P4	35	Pathologic pregnancy	Azitromycin, cefixime (urinary tract infection)	No	Gestational diabetes	NA ^b^	Yes	Metronidazole	No	Day −159/+12	Unknown ^c^
P5	36	Diarrhea	No	No	Gestational diabetes	No	Yes	Metronidazole	No	Day −197/+13	001
P7	27	Pathologic pregnancy	Clindamycin, cefotaxime (PRM ^a^)	Yes	Gestational diabetes PRM ^a^	No	No	Metronidazole	No	Day −8/+3	JV14

^a^ PRM: premature rupture of membranes. ^b^ NA: data non available. ^c^ PCR: ribotyping was unknown.

## Data Availability

The original contributions presented in this study are included in the article. Further inquiries can be directed to the corresponding author.
